# Review and Analysis of Electrochemical Instrumentation Design for Continuous Multi-Analyte Microfluidic Sensor Arrays

**DOI:** 10.3390/s26175334

**Published:** 2026-08-23

**Authors:** Samuel Lobert, Zahid Rashid Sheikh, Navid Yazdi, Derek Goderis, Andrew J. Mason

**Affiliations:** Department of Electrical and Computer Engineering, Michigan State University, East Lansing, MI 48824, USA; lobertsa@msu.edu (S.L.); sheikhz3@msu.edu (Z.R.S.);

**Keywords:** review, electrochemical sensing, potentiostat, microelectrode array, simultaneous multi-technique sensing, crosstalk, microfluidics, lab-on-chip, continuous sensors, multi-analyte sensors, electrochemical instrumentation, point-of-care biosensors

## Abstract

Electrochemical sensor arrays that perform simultaneous multi-technique (SMT) measurements within a shared electrolyte are essential for continuous, multi-analyte detection in microfluidic platforms for environmental and healthcare monitoring. This review examines the potentiostat architectures and electrode geometries relevant to SMT operation. Traditional single channel and multi-electrode potentiostat topologies are surveyed, and their suitability for multi-cell shared-electrolyte environments is evaluated. Additionally, crosstalk mechanisms in shared electrolytes are classified into chemical, electrical, and a newly identified category termed stability-based interference, which arises from conflicting feedback loops in conventional grounded working electrode instrumentation. A survey of existing multi-cell platforms reveals that most reported systems either avoid true SMT operation or address crosstalk primarily through electrode geometry without systematic evaluation of instrumentation effects. Based on this analysis, we introduce an instrumentation and electrode geometry co-design framework that provides a unified design pathway toward continuous multi-analyte microfluidic sensors for wearable and point-of-care applications.

## 1. Introduction

Electrochemical sensing is a powerful technique for environmental and health monitoring applications. Within the broader landscape of sensing technologies, electrochemical methods have gained widespread adoption due to their ability to directly transduce biological and chemical processes into quantifiable electrical signals [1]. Electrochemical techniques are also highly versatile, with applications across multiple sensing domains, enabling the detection and quantification of solid, liquid, gaseous, and biological analytes [2]. In environmental sensing applications, electrochemical methods have been shown to provide a detection strategy for airborne particulate matter. Techniques such as single-entity electrochemistry enable the selective chemical identification of harmful particulate matter species, including black carbon [3,4] and heavy metal nanoparticles [5,6]. Separately, a variety of electrochemical bioaerosol sensing approaches have been developed to monitor a broad range of indoor airborne pollutants [7,8]. For health monitoring applications, electrochemical biosensors are used to enable the detection and screening of numerous cancer biomarkers [9,10,11,12,13] and a multitude of neurotransmitters, among other biomarkers with key significance to analyzing human health [14]. Importantly, electrochemical sensing methods are highly beneficial because they enable sensitive and selective sensing systems [2]. By varying the electrode material [15,16,17], or by adding an electrode functionalization layer [7,18,19,20], electrochemical sensors can become highly selective toward individual analytes of interest.

As these sensors mature from laboratory demonstrations, there is a growing need to package the sensing technologies in miniaturized platforms to provide point-of-care (PoC) and wearable systems [10]. PoC electrochemical sensors provide low-cost and easy-to-use systems for at-home diagnoses [21], and wearable sensors can provide continuous data with high spatial-temporal resolution to allow individuals to acutely monitor their health [3]. Microfluidic devices provide a platform on which electrochemical sensors can be implemented for PoC and wearable applications. Microfluidics supports miniaturization while maintaining analytical performance. Across both environmental and health monitoring domains, the advantages of microfluidic integration converge on the ability to achieve continuous, multi-analyte sensing. Continuous operation reduces required sample volumes, enables rapid analysis, and provides real-time data streams suitable for longitudinal monitoring in wearable and PoC systems [22,23]. Multi-analyte capability enables correlation of biomarkers, simultaneous monitoring of diverse environmental exposures, and cross-validation of sensing modalities within the same sample volume.

Realizing the full potential of continuous, multi-analyte, microfluidic sensors requires simultaneous multi-technique (SMT) operation, in which independently biased electrochemical cells operate concurrently within a shared electrolyte. Despite the benefits of continuous SMT sensors, most reported electrochemical systems rely on time-division multiplexing, where measurement techniques are applied sequentially using single-channel potentiostat instrumentation [24,25,26,27,28,29,30]. While multiplexing simplifies system implementation, it fundamentally limits temporal resolution and yields only pseudo-continuous measurements. These limitations are particularly significant in electrochemical systems, where measurement times are often many orders of magnitude slower than the capability of electronic circuits in measurement instrumentation. Sequential operation therefore introduces settling delays between measurements [2,31] and scales poorly with increasing channel count. For instance, acquiring a single frame from a 200-pixel electrochemical imaging array can require on the order of tens of minutes, whereas fully parallel measurements reduce acquisition times to seconds [32]. Consequently, realizing true continuous multi-analyte sensing necessitates SMT instrumentation capable of fully parallel operation, with each channel optimized for its target analyte.

The primary challenge in achieving SMT operation arises from the shared microfluidic electrolyte, which forms a continuous conductive medium between all sensing electrodes. Although highly conductive supporting electrolytes are required to maintain diffusion-controlled electrochemical reactions [33], they also introduce unintended electrical and chemical coupling between neighboring cells. When electrodes are independently biased, these interactions give rise to crosstalk, perturbing the reactions on both cells, and ultimately limiting independent operation within the same sensing volume. These interaction effects cannot be mitigated through straightforward extension of conventional potentiostat architectures. Standard single-channel designs assume an isolated electrochemical cell, and extending them to multi-electrode systems without accounting for shared-electrolyte coupling leads to measurement error and instability [2,34]. As a result, enabling continuous, multi-analyte SMT sensing requires a fundamentally different design paradigm in which electrochemical sensor arrays and instrumentation are co-designed to manage coupling within the system.

Existing literature addresses components of this challenge in isolation. Reviews of electrochemical biosensors and multiplexed platforms primarily emphasize biochemical selectivity, assay development, and lifespan of wearable sensors [9,22,24,27,35], while reviews of potentiostat circuit architectures focus on low-voltage CMOS design, integration strategies, and measurement performance, often assuming a single working electrode or neglecting shared-electrolyte effects [1,36,37,38,39]. Studies of crosstalk in electrode arrays typically isolate individual mechanisms such as capacitive coupling [40,41,42], shared-resistance coupling [43,44], or shielding approaches [45], and are often limited to single-technique operation.

What remains lacking is a unified framework that considers potentiostat circuit architecture and microelectrode array fabrication within a coupled design space to address interaction effects in SMT-enabled systems. This review addresses the gap by providing the first analysis that bridges the interaction between electrochemical instrumentation and adjacent electrochemical reactions connected in a conductive liquid electrolyte. Moreover, we present findings to support the argument that achieving interaction-aware SMT operation in a shared electrolyte requires co-design across instrumentation and fabricated sensor geometry, and we identify ideal combinations of instrumentation and electrode geometry that would enable true SMT operation. Section 2 establishes the electrochemical foundations for sensing. Section 3 surveys single-channel potentiostat architectures. Section 4 categorizes electrical and chemical coupling mechanisms in shared electrolytes. Section 5 reviews circuit- and system-level mitigation strategies, and Section 6 synthesizes these insights into a co-design framework for continuous multi-analyte sensors. Finally, Section 7 outlines open challenges and future directions toward realizing fully continuous, multi-analyte microfluidic sensing platforms.

## 2. Electrochemical Foundations for SMT Sensors

**Figure 1:**
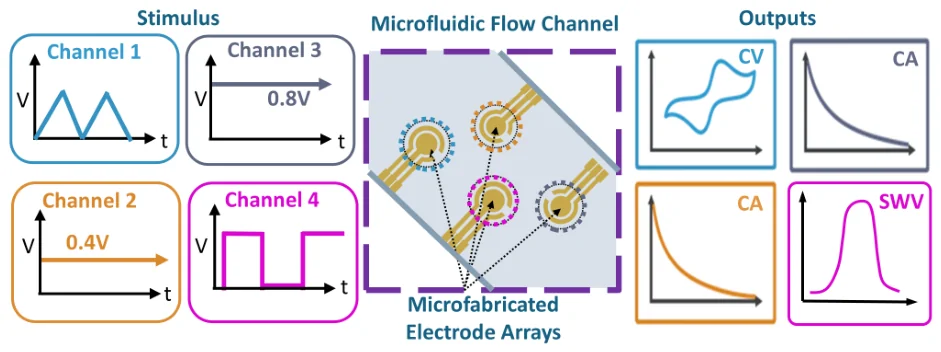
Conceptual illustration of simultaneous multi-technique (SMT) electrochemical sensing on a microfabricated electrode array within a shared microfluidic flow channel. Four independent potentiostat channels apply distinct stimulus waveforms in parallel to adjacent three-electrode cells: cyclic voltammetry on channel 1, chronoamperometry at a constant 0.4 V bias on channel 2, chronoamperometry at a constant 0.8 V bias on channel 3, and square-wave voltammetry on channel 4. Each channel records its technique-specific current response simultaneously and independently, enabling continuous multi-analyte detection within a single shared electrolyte.

Multi-analyte sensing requires adjacent electrochemical cells to be biased at different potentials, often using different measurement techniques, while simultaneously immersed in a common, highly conductive electrolyte. A conceptual representation of SMT operation on a microfabricated electrode array is shown in Figure 1, in which multiple potentiostat channels apply distinct stimulus waveforms in parallel to adjacent three-electrode cells within a shared microfluidic flow channel and simultaneously record technique-specific current responses. Each of these requirements is individually well understood in single-cell electrochemistry, but their simultaneous enforcement in a shared volume is precisely what produces the coupling mechanisms that motivate the co-design framework developed in this review. This section is therefore included to review the fundamental electrochemical sensing principles required to extend single-cell measurements to SMT measurements. For a further overview of electrochemical theory, the reader is directed towards [33,46,47,48,49].

### 2.1. Analyte-Specific Redox Potentials as the Basis for Multi-Analyte Sensing

Electrochemical sensing exploits the fact that each redox-active analyte undergoes oxidation or reduction at a characteristic electrode potential. A redox couple is expressed as

(1) $$\mathrm{Ox}+ne^{-}⇌\mathrm{Red}$$

where Ox and Red denote the oxidized and reduced species and *n* is the number of electrons transferred. The potential at which this reaction proceeds at equilibrium is governed by the Nernst equation,

(2) $$E=E_{0}-\frac{RT}{nF}\ln\;\left(\frac{\left[\mathrm{Ox}\right]}{\left[\mathrm{Red}\right]}\right)$$

which links the electrode potential to the standard potential $E_{0}$ of the couple and the activities of the participating species [33].

The practical consequence of Equation (2) for SMT sensing is that distinct analytes generally require distinct electrode bias conditions to be selectively interrogated [4,33,50]. A single-potential measurement is therefore incompatible with the continuous, multi-analyte operation targeted in this work. Achieving simultaneous detection of multiple species in a shared microfluidic volume requires that neighboring working electrodes be held at different potentials at the same time: a condition that is trivial to satisfy for isolated cells but becomes the root cause of electrical coupling when those cells share an electrolyte.

### 2.2. Electroanalytical Techniques and Analyte Specific Waveforms

The requirement for independent electrode bias extends beyond static potentials. Different electroanalytical techniques apply distinct time-varying waveforms to the working electrode, each optimized for a particular class of information. In amperometric techniques such as chronoamperometry (CA), a constant potential (or current) is applied, and the resulting current (or voltage) is recorded as a function of time [1,36]. Under semi-infinite linear diffusion, the steady-state current is proportional to analyte concentration, providing a quantitative readout. In voltammetric techniques such as cyclic voltammetry (CV), square-wave voltammetry (SWV), or differential pulse voltammetry (DPV) the applied potential is swept or modulated and the current is recorded as a function of potential, yielding information about analyte concentration, kinetics, reversibility, and the presence of multiple analytes [49,51,52].

The measured current at any electrode is the sum of a faradaic component, arising from charge transfer associated with the redox reaction of interest, and a non-faradaic component arising from charging and discharging of the interfacial double layer. Because different techniques impose different waveform profiles, the relative contribution of these two components varies substantially between techniques. Importantly, pulse and sweep methods inherently produce non-faradaic currents caused by continuously distributing charge on the double-layer capacitance that forms on electrodes [33]. This matters for SMT operation because adjacent cells running different techniques will generate technique-specific current profiles, which must be independently controlled and measured without mutual interference.

From the standpoint of the reaction-driving electrical instrumentation, this establishes the fundamental difference of SMT operation. The potentiostat must not only establish adjacent working electrodes at distinct DC bias points, but it must also be able to execute arbitrary, independent excitation waveforms and provide parallel measurements for each stimulus channel. The remainder of this review treats this combined requirement of independent potentials or independent techniques, operating concurrently in a shared electrolyte, as the defining constraint on SMT system design.

### 2.3. The Conductive Supporting Electrolyte Shared Among Cells

Electrochemical sensing provides an interfacial measurement as the applied potential at an electrode decays exponentially into solution over the Debye length which is typically 1–10 nm in aqueous electrolytes of moderate ionic strength [53,54]. Maintaining this short screening length requires a sufficient concentration of mobile ions in solution.

The same mobile ions also ensure that mass transport to the electrode is dominated by diffusion rather than migration. From the Nernst–Planck equation,

(3) $$J_{i}\left(x\right)=-D_{i}\frac{dC_{i}\left(x\right)}{dx}-\frac{z_{i}F}{RT}D_{i}C_{i}\left(x\right)\frac{d\Phi (x)}{dx}-C_{i}\left(x\right)v\left(x\right),$$

the migration term couples analyte flux to the local solution field, and therefore to the potentials of neighboring electrodes. An inert supporting electrolyte at sufficient concentration screens these fields and fixes the bulk conductivity, making diffusion-controlled quantitative measurement possible [33]. Although some sensing regimes operate at reduced ionic strength [55,56], a majority of sensing applications prefer the addition of a highly conductive supporting electrolyte [3,7,19].

### 2.4. Randles Equivalent Circuit and Inter-Cell Coupling

Voltammetric measurements are typically performed using a three-electrode configuration consisting of a working electrode (WE), reference electrode (RE), and counter electrode (CE) for increased measurement stability over a two-electrode configuration. The potentiostat controls the electrochemical cell potential ($V_{\mathrm{cell}}$) by enforcing a bias between the WE and RE while preventing current flow through the RE, so that the RE’s potential remains stable. The CE sources or sinks the current required to maintain $V_{\mathrm{cell}}$ and to support the complementary electrochemical reaction, ensuring charge balance [33].

**Figure 2:**
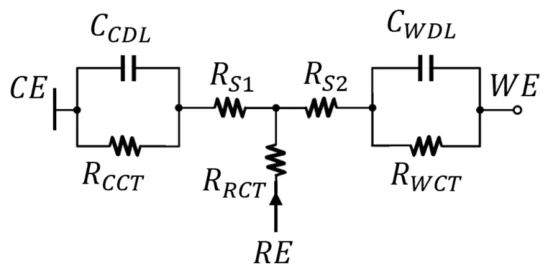
Generic Randles equivalent circuit model of a three-electrode electrochemical cell, adapted from [57]. The solution resistances ($R_{s1}$, $R_{s2}$) represent conductive paths through the electrolyte between electrodes. In SMT systems, analogous resistive pathways exist between electrodes of adjacent cells within a shared electrolyte, enabling unintended signal coupling and crosstalk.

The three-electrode cell can be modeled by the Randles equivalent circuit. A circuit model for the generic three-electrode cell, adapted from [57], is shown in Figure 2. Here, $R_{CCT}$, $R_{RCT}$, and $R_{WCT}$ denote the charge-transfer resistances at the counter, reference, and working electrodes; $C_{CDL}$ and $C_{WDL}$ are the corresponding double-layer capacitances; and $R_{S1}$ and $R_{S2}$ are the solution resistances between electrodes through the electrolyte. More detailed models are required to capture material-, functionalization-, and frequency-dependent interfacial behavior [58,59,60].

The key observation for SMT design is that the $R_{S}$ elements are not local cell properties but localized representations of a continuous conductive medium. In a shared microfluidic volume, analogous resistances exist between the electrodes of every cell due to the electrolyte [43,61]. With neighboring active cells the solution resistance carries currents outside the intended measurement loop and results in a coupled network in which the processes at one cell are measured by its neighbor.

## 3. Potentiostat Architectures

A potentiostat is the instrumentation that enforces a controlled potential between the working electrode (WE) and reference electrode (RE) while measuring the resulting current flowing through the electrochemical cell [1,36,37]. Single-channel potentiostats are commonly categorized by which electrode is held at a stable DC potential known as analog ground. This classification yields three traditional topologies: grounded-WE (GWE), grounded-RE (GRE), and grounded-CE (GCE) potentiostats. Here, ground refers to a DC reference voltage rather than a physical connection to 0 V. For potentiostats required to measure both anodic and cathodic currents on a single power supply, this node is often set at mid-supply to enable bidirectional current measurement [34,62].

### 3.1. Grounded Working Electrode Potentiostat

**Figure 3:**
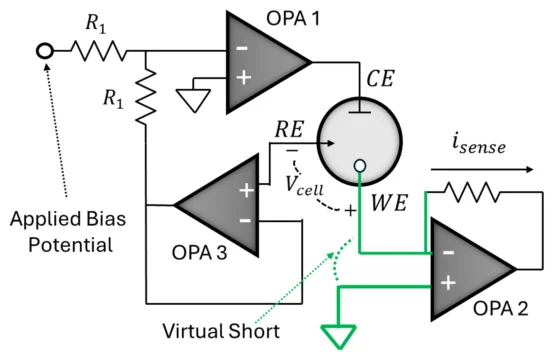
Traditional GWE potentiostat topology with transimpedance amplifier current readout.

The traditional, and most commonly depicted, potentiostat topology is the grounded working electrode (GWE) topology, shown in Figure 3. This topology is widely described in fundamental electrochemistry texts and embedded instrumentation references [2,63,64,65,66,67,68,69,70,71,72,73,74,75,76,77]. This topology, as well as the others, contains two main subcircuits: the potential biasing circuitry and the current measurement circuitry. The electrochemical current is measured using an operational amplifier (OPA)-based transimpedance amplifier (TIA) connected to the WE. The virtual short between the OPA inputs causes the WE to be driven to the stable DC analog ground potential connected to the non-inverting input.

To generate the desired cell potential, OPA 2 in Figure 3 senses the RE potential and adds it to the applied bias voltage through an adder stage implemented with OPA 3 and matched resistors. OPA 2 drives the RE node to the applied bias potential, which establishes $V_{cell}=V_{WE}-V_{RE}$, and it prevents current from flowing through the RE because the RE is connected to its high-impedance input. The CE is allowed to float to whatever potential is required to supply or sink the electrochemical current and to overcome the solution impedance between the CE and RE in the electrolyte.

This topology is suitable for most electrochemical cells because the current is measured at the working electrode, which is the electrode where the reaction of interest occurs. In addition, holding the WE at a virtually grounded potential reduces noise in the measurement path and supports high-precision current readout. The potentiostat architectures described here represent the traditional operational amplifier-based implementations that employ transimpedance amplifiers for current readout. Although alternative potentiostat designs exist that do not conform to these grounded-electrode topologies, the TIA-based configurations remain the most commonly adopted approach in PCB-based embedded systems and form the focus of this work [1,36].

### 3.2. Grounded Reference Electrode Potentiostat

**Figure 4:**
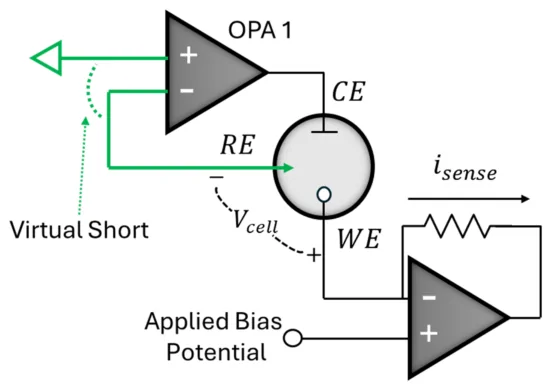
Traditional GRE potentiostat topology with transimpedance amplifier current readout.

The grounded reference electrode (GRE) topology shares many similarities with the GWE topology, but as shown in Figure 4, the main difference is that both current readout and cell biasing are performed on the WE while the RE is held at a stable analog ground.

Although the two topologies appear nearly identical from a circuit perspective, holding the RE at a fixed potential rather than varying it can significantly affect measurements that require non-DC biasing, such as cyclic voltammetry or square wave voltammetry. A prominent case arises in experiments involving multiple working electrodes or multiple three-electrode cells [78,79,80].

One benefit of the GRE potentiostat is that it allows for an increased range in applied bias potentials over the GWE circuit. Placing the RE at an analog ground equal to half the supply voltage enables bidirectional current measurement and provides rail-to-rail cell bias capability, which is twice that of the GWE and grounded counter electrode (GCE) topologies [62].

### 3.3. Grounded Counter Electrode Potentiostat

**Figure 5:**
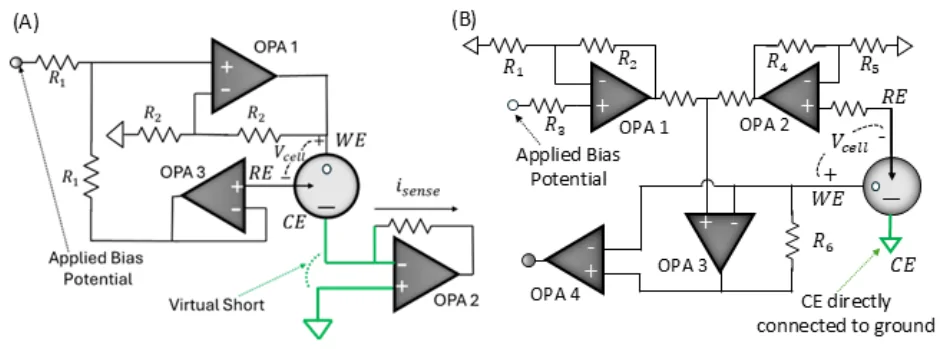
GCE potentiostat topologies. (**A**) Conventional implementation in which the CE is held at a virtual ground by an OPA-based TIA, with current measured at the CE. (**B**) Implementation of GCE potentiostat in which the CE is physically connected to 0 V. The WE is driven relative to a floating RE and current is measured at the WE through a current follower [83,84].

The grounded counter electrode (GCE) topology follows the trend of placing one electrode at a stable potential, with two distinct implementations shown in Figure 5A,B that differ in how the CE is grounded. In the most common implementation (Figure 5A), the CE is held at a virtual ground by an OPA-based TIA, and the cell current is measured at the CE node. A full derivation of the circuit is given in [37,78]. This implementation is useful when measuring at the WE is not ideal, for example when the TIA is capacitively loaded by large double-layer capacitances during supercapacitor analysis [81,82]. It was also originally proposed for measurements with conductive reservoirs, where connecting the WE to the low-impedance output of an OPA rather than to a high-impedance input reduced electromagnetic interference and high-frequency measurement errors [78].

The second implementation (Figure 5B) replaces the virtual ground with a direct physical connection to 0 V. It has similarly been used for metal-walled reactors and other systems where the cell body cannot be isolated from earth ground [83,84]. The WE is biased relative to a floating RE by adding the applied potential to the measured RE voltage, and current is measured at the WE through a current follower where the CE is part of the grounded reactor wall.

### 3.4. Fully Differential Potentiostats

The three grounded-electrode topologies described above all use single-ended signaling where one electrode is held at analog ground, and all potentials and currents are referenced to that node. A distinct family of fully differential potentiostats instead uses differential signaling along part or all of the signal path, motivated primarily by improved noise immunity and dynamic range in low-voltage CMOS implementations. Two main architectural variants appear in the literature.

**Figure 6:**
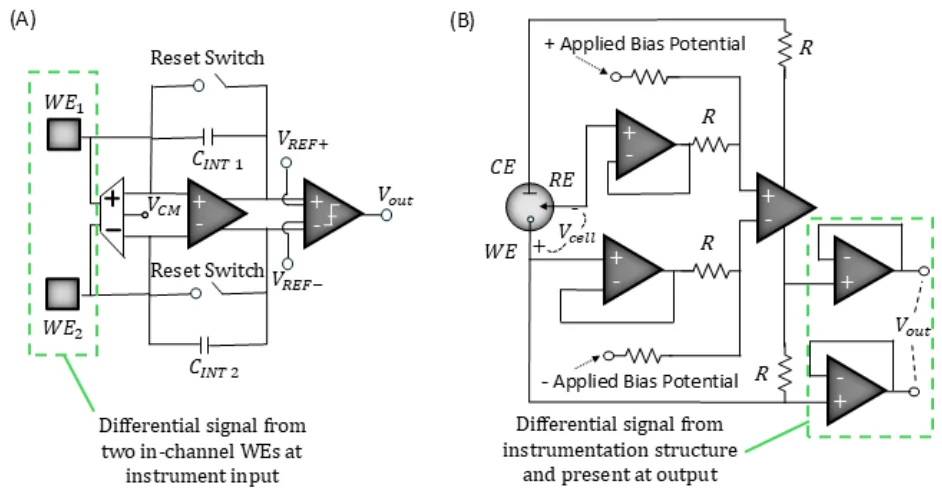
Fully differential potentiostat architectures. (**A**) Differential-WE configuration in which two matched on-chip working electrodes share the same electrolyte and the differential current acquisition circuit rejects common-mode disturbances. Adapted from [85]. (**B**) Single-WE configuration with a fully differential transimpedance stage producing a differential output for doubled signal swing. Adapted from [1,86].

The first variant, shown in Figure 6A, extends differential signaling to the sensor interface itself by pairing the active WE with a second, nominally identical WE exposed to the same electrolyte. The differential current acquisition circuit measures the difference between the two electrode currents, suppressing any common-mode disturbance, including double-layer charging currents, solution-borne noise, and common-mode potentiostat noise [85].

The second variant, shown in Figure 6B, retains a single WE but replaces the single-ended transimpedance stage with a fully differential transimpedance amplifier producing a differential output voltage. Because the differential output swings symmetrically about a common-mode reference, the available signal range is approximately doubled for the same supply voltage, and the differential signal path inherently cancels even-order harmonic distortion to improve linearity at low input currents [1,86].

## 4. Challenges in Shared-Electrolyte SMT Arrays

When multiple electrochemical cells operate simultaneously in a shared microfluidic volume, the single-cell assumptions underlying standard electrochemical analysis may no longer hold due to interaction effects between adjacent cells. These interaction effects manifest as crosstalk, in which signal originating at one cell is measured at its neighbor. Crosstalk in SMT electrochemical measurements can be broadly separated into two categories: chemical and electrical. Chemical crosstalk arises when depletion regions and analyte concentration gradients overlap between adjacent cells, while electrical crosstalk arises from gradients in electrical potential between cells in a shared solution and from shared paths within the measurement instrumentation itself. In practice, multiple mechanisms typically operate concurrently, and both categories must be characterized before effective mitigation strategies for SMT measurements can be developed.

### 4.1. Chemical Crosstalk

Chemical crosstalk occurs when redox reactions at adjacent WEs consume reactants and accumulate products faster than diffusion or convection can replenish them, producing depletion regions that grow outward and eventually overlap [12,87,88,89,90,91]. The geometry of the depletion region depends on the electrode size relative to the diffusion layer thickness. At a macroelectrode, where the electrode dimension greatly exceeds the diffusion layer, transport is dominated by planar one-dimensional diffusion, producing the familiar peak-shaped curve in CV measurements. At a microelectrode, the depletion-region thickness rapidly becomes comparable to the electrode dimension, and mass transport transitions from planar to radial, producing a steady-state sigmoidal CV with a limiting-current plateau [33,87].

**Figure 7:**
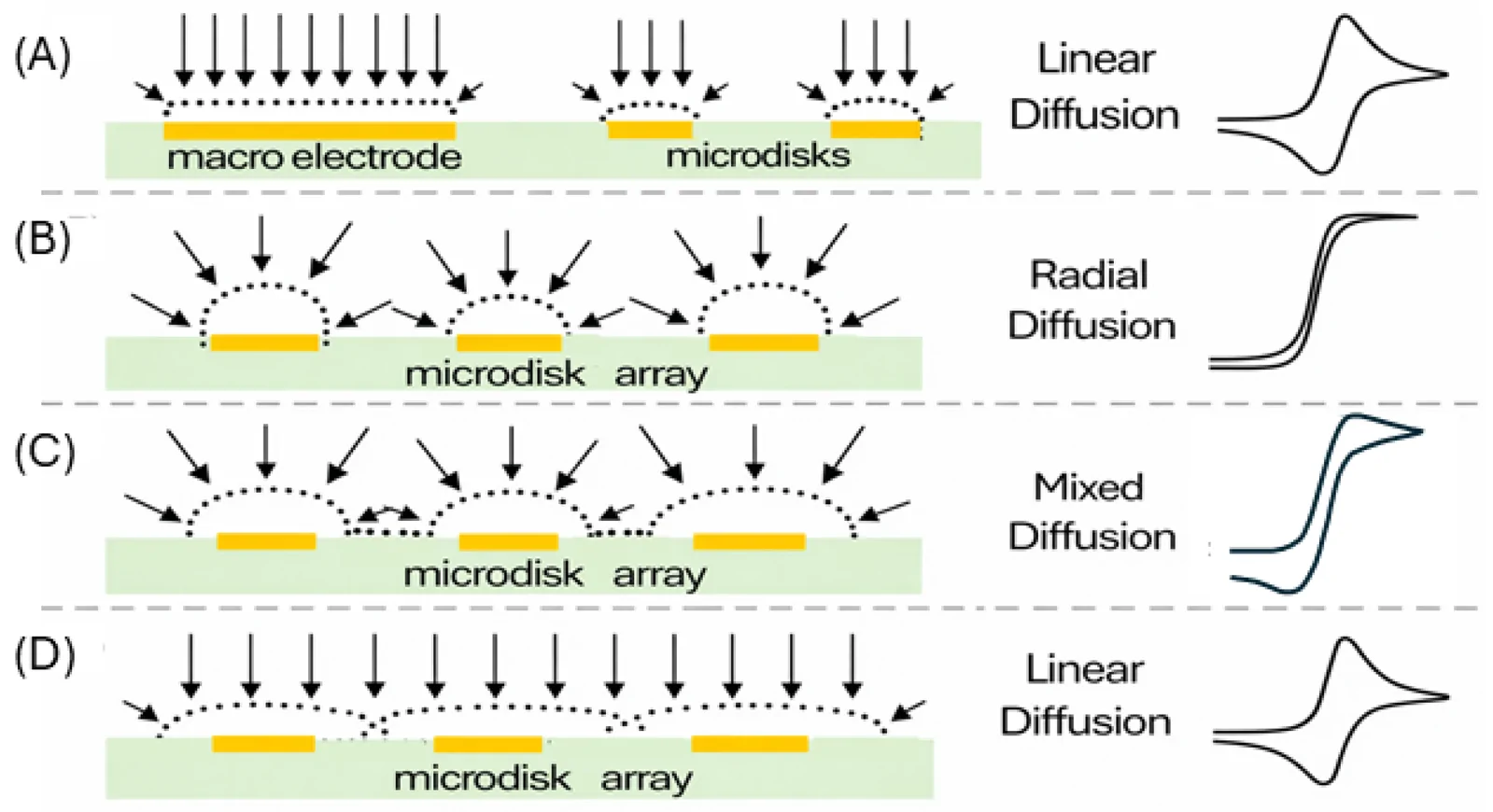
Evolution of depletion regions in a microelectrode array leading to chemical crosstalk. (**A**) Linear diffusion at a macroelectrode and at microdisk electrodes under startup conditions; (**B**) steady-state radial diffusion at each microdisk; (**C**) partial overlap of depletion regions producing mixed radial/linear behavior; (**D**) complete overlap, at which the array responds as a single macroelectrode. Adapted from [33,90].

When the depletion regions of adjacent electrodes overlap, the analyte flux to each electrode is reduced because its neighbor has already partially depleted the local solution, and, more significantly, the array begins to behave electrochemically as a single macroelectrode rather than as a set of independent sensing sites. As shown in Figure 7, the radial-diffusion-enhanced sigmoidal response reverts toward the peak-shaped response characteristic of linear diffusion, and the spatial resolution advantage of a high-density microelectrode array is lost [90,92,93,94,95].

This conventional framing, however, applies primarily to working electrode arrays held at the same potential, without continuous flow, while probing a single analyte. Continuous SMT systems introduce additional considerations. When adjacent cells target different, non-interacting analytes, overlapping depletion regions may have negligible analytical effect because each electrode consumes a different species. More problematically, however, a product generated at one electrode may be transported to a neighboring electrode biased at a different potential and undergo an unintended secondary reaction there. This would generate an unintended faradaic current that is indistinguishable from the target signal of the neighboring cell. Accounting for both the absence and the presence of inter-analyte coupling is therefore essential to correctly interpret SMT measurements, and the relevant analysis depends on the specific redox chemistry of each target analyte rather than on geometry alone.

### 4.2. Electrical Crosstalk

Electrical crosstalk arises whenever shared electrical paths violate the single-cell conditions assumed in voltammetric or amperometric analysis. Electrical crosstalk can appear through multiple mechanisms such as capacitive coupling between signal leads and electrode structures, resistive coupling through the shared electrolyte, and coupling through shared instrumentation grounds or supply paths [29,44,45]. These mechanisms differ in their dependencies on electrode geometry, electrolyte conductivity, and instrumentation design, and they typically act in combination rather than in isolation.

#### 4.2.1. Capacitive Electrode and Lead Coupling

Capacitive crosstalk arises from parasitic capacitances between adjacent electrode leads and, in some geometries, between the electrodes themselves. This mechanism has been extensively characterized in high-density microelectrode arrays for neural recording, where decreasing electrode dimensions and increasing array density compress signal routing into close proximity, increasing the parasitic capacitance between neighboring lines [40,41,42,96,97]. Because the impedance of a parasitic capacitor decreases with frequency, capacitive coupling becomes most consequential at the higher signal bandwidths characteristic of neural recording, typically 300 Hz to 1 kHz [98].

For SMT microfluidic sensors, the relevance of capacitive coupling depends on the bandwidth of the applied excitation. Low-frequency techniques such as traditional cyclic voltammetry have signal excitation frequencies in the millihertz to hertz regime, whereas higher-bandwidth techniques such as fast-scan cyclic voltammetry, square-wave voltammetry, and electrochemical impedance spectroscopy can be appreciably affected, particularly when adjacent channels carry signals at different frequencies or phases.

#### 4.2.2. Inter-Cell Coupling Through the Solution Resistance

The second and most consequential mechanism for SMT microfluidic sensors is resistive coupling through the shared electrolyte itself. Section 2.4 established that the solution-resistance elements of a single-cell Randles model are localized representations of a continuous conductive medium. In a shared microfluidic volume, this generalizes to a matrix of inter-electrode solution resistances connecting every electrode of every cell to every other electrode through the electrolyte [43,99,100]. When adjacent cells are biased at different potentials, as required for multi-analyte sensing per Section 2.1, voltage gradients develop across the solution’s resistive matrix, and currents flow between electrodes of different cells along paths that were never part of the intended single-cell measurement loop.

**Figure 8:**
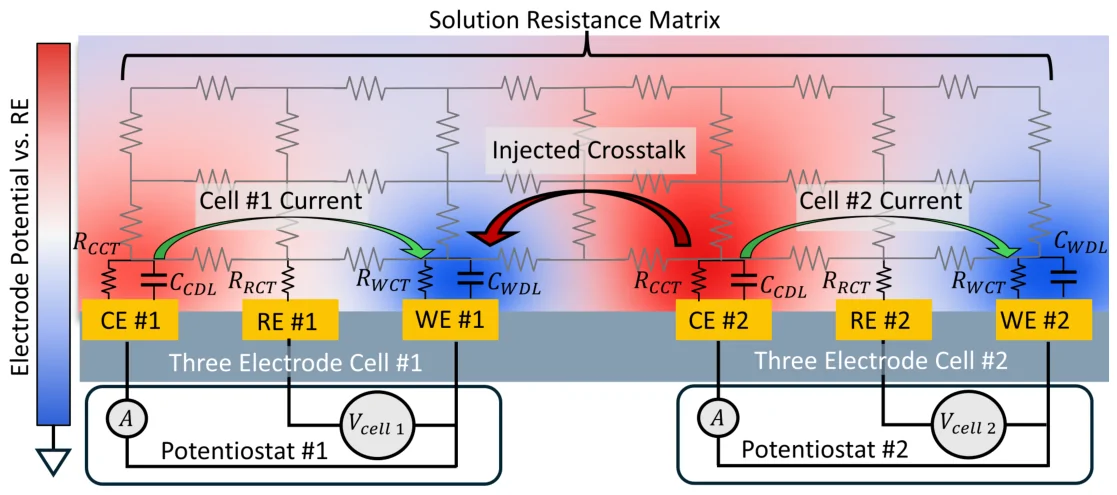
Two adjacent three-electrode cells in a shared microfluidic volume, modeled as a network of inter-electrode solution resistances. The on-cell solution resistances ($R_{s1}$, $R_{s2}$) of the single-cell Randles model are extended to a network of solution resistances throughout the 3D conductive channel volume. When the two adjacent cells are held at different potentials, electrical crosstalk is caused from the inter-cell potential gradient.

Vesztergom et al. [43,99] analyzed this coupling mechanism in beaker-scale rotating ring-disk electrode (RRDE) systems, in which two working electrodes share a common solution path with a single reference and counter electrode. Through combined numerical simulation and experimental validation, they identified three properties that govern crosstalk severity. First, crosstalk primarily affects the electrode with the higher interfacial impedance, since the same induced potential disturbance produces a larger relative current error at the higher-impedance electrode. Second, crosstalk magnitude grows with the value of the shared solution resistance in the common current path between the two working electrodes. Third, crosstalk becomes increasingly apparent when high sweep rates or excitation frequencies are applied, because the larger transient currents associated with fast perturbations produce correspondingly larger uncompensated potential shifts across the shared resistance. The same mechanism has been independently documented in scanning electrochemical microscopy [101], in flow-based multichannel electrochemistry [80], in integrated biosensor arrays with shared reservoirs [44], and as volume-conduction coupling in high-density neural probes operating in cerebrospinal fluid [45,95], see Figure 8.

#### 4.2.3. Instrumentation Ground Loops

A third electrical crosstalk mechanism arises from the potentiostat instrumentation itself, independent of the electrolyte coupling discussed above. In an ideal transimpedance amplifier (TIA), the inverting input is held at a virtual ground potential through negative feedback. In practice, finite open-loop gain, limited bandwidth, and large transient input currents cause deviations from this ideal condition, such that the effective ground potential at the working electrode node shifts transiently in response to the measured current.

When multiple potentiostat channels share a common ground plane, power return path, or reference node, these small ground potential variations propagate through the shared conductive paths on the printed circuit board. A current transient in one channel can therefore produce a measurable voltage offset in adjacent channels, manifesting as inter-channel crosstalk that is unrelated to the electrochemical cell itself. This mechanism becomes more significant when high-gain TIAs are used, when large capacitive double-layer charging currents are present at the onset of voltammetric scans, or when multiple potentiostat front-ends share a common supply and ground reference [102,103]. Ground loops can also form at the system level when two instruments reference ground at physically different points but are electrically connected through both signal cables and earth ground, allowing unintended circulating currents to flow through the resulting closed loop. To address this issue, many commercial potentiostat suppliers offer galvanically isolated instruments for simultaneous measurements [104,105], although galvanic isolation does not address the other electrical crosstalk mechanisms discussed above.

## 5. Circuit and System Approaches to Simultaneous Multi-Technique Operation

Given the coupling mechanisms identified in Section 4, many reported approaches favor multiplexed sensing, which bypasses the described challenges but fails to meet the requirements of continuous multi-analyte microfluidic sensors. Several systems in the literature, while not demonstrating fully continuous multi-analyte operation, provide methods that, when combined, may enable successful SMT electrochemical measurements. As these systems were not specifically designed for SMT measurements, it is critical to identify the failure modes that are present in each system in order to support future instrumentation with SMT compatibility.

### 5.1. Simultaneous Single-Technique Arrays

A partial step toward simultaneity is the simultaneous single-technique (SST) array, in which all cells are biased at the same potential or execute the same waveform simultaneously, and each cell has a dedicated current-readout channel [103,106,107]. Because all cells are driven at identical potentials, no voltage gradient develops across the shared electrolyte, and shared volume–conductor coupling is suppressed by construction. Minor crosstalk can still arise when individual reactions at each cell produce slightly different local potentials on adjacent electrodes, but the use of an identical stimulus signal across all cells minimizes these interaction effects. SST arrays have been used with great success in high-density applications where spatial resolution is critical, including electrochemical imaging with dense pixel arrays, simultaneous neurochemical recording across neural probes [108], and amperometric imaging of redox species [32]. As pixel densities increase towards $10^{4}$–$10^{5}$ electrodes per ${\mathrm{mm}}^{2}$, fully parallel readout becomes area- and power-prohibitive, and partial multiplexing is typically reintroduced [109].

The critical limitation of SST arrays is that they provide no independent electrochemical control per cell. Every WE runs the same technique at the same potential, allowing SST arrays to image a single analyte spatially but not to distinguish multiple analytes using different techniques in parallel, which is the goal of SMT. From a circuit perspective, SST implementations typically use the traditional grounded-electrode potentiostat topologies described in Section 3, replicated across each pixel or channel. In SST, all cells share an identical stimulus, so these topologies operate within their intended single-cell regime and do not encounter the failure modes that arise when adjacent cells are biased independently. The following section examines how the same traditional topologies behave when extended to SMT operation, where independent biasing introduces interactions that the single-channel architecture was never designed to handle.

### 5.2. Extending the Grounded-Electrode Topologies to SMT

A natural question is whether the established single-channel grounded-electrode topologies can be replicated *N* times to build an *N*-channel SMT instrument. The answer depends strongly on how each traditional single-channel potentiostat circuit behaves when multiple instruments operate on adjacent electrode sets in a shared conductive solution. The following subsections examine the three grounded-electrode topologies in turn, together with the bipotentiostat and multi-WE architectures derived from the GWE structure, and trace a common thread: each successive topology addresses the instrumentation-level stability-based interference of the direct GWE replication, yet electrical crosstalk through the shared solution resistance persists until the electrode geometry is brought into the design.

#### 5.2.1. Grounded-WE Extensions and Stability-Based Interference

**Figure 9:**
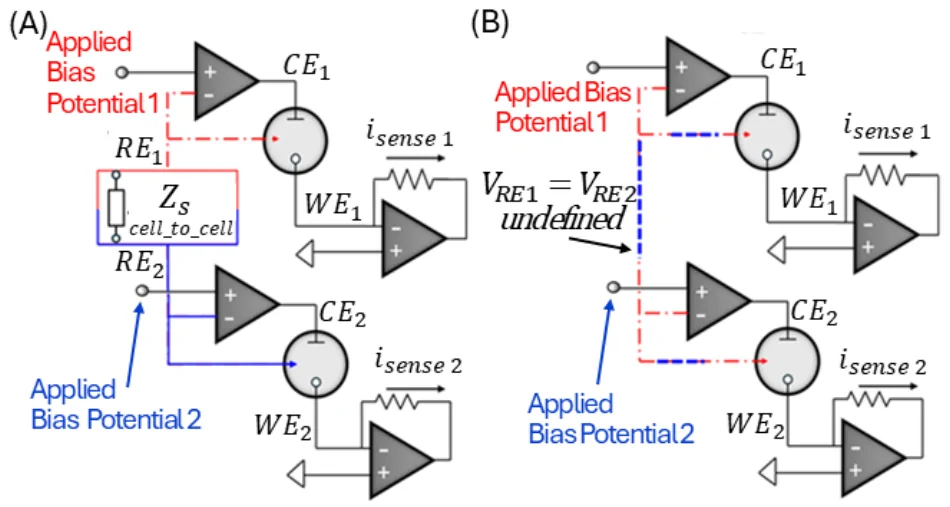
Stability-based interference in dual grounded-WE potentiostats operating in a shared electrolyte. (**A**) When each cell has an independent RE, the cell potentials are coupled through the inter-cell solution impedance $Z_{s}$ and current flows through each RE. (**B**) When the cells share a common RE, the two biasing feedback loops attempt to drive the same node to different potentials, leaving the cell bias undefined.

The GWE topology is the default choice for SMT extension because it dominates single-channel practice (Section 3.1). Replicating a GWE potentiostat for SMT operation in a shared electrolyte, however, causes the operational amplifiers that implement the biasing circuitry to become unstable [2]. This failure mode manifests in two related forms depending on the reference electrode configuration, illustrated in Figure 9.

When each cell employs an independent RE, the reference nodes are electrically coupled through the shared solution resistance. If different stimulus potentials are applied simultaneously, a potential difference develops across $R_{s}$ and drives current between the REs. This violates the fundamental three-electrode requirement that negligible current flow through the RE, and the RE potential is no longer defined strictly by electrochemical equilibrium but is influenced by instrumentation-induced coupling. Current flow through the RE can also accelerate its degradation and reduce overall sensor lifetime [110]. Increasing the inter-cell distance would similarly increase $R_{s}$, but this conflicts directly with the miniaturization requirements of microfluidic and lab-on-chip platforms.

When the two cells share a common RE, $R_{s}$ effectively collapses to a short circuit, and the failure mode shifts from current injection to a feedback-loop conflict. Multiple bias amplifiers attempt to enforce different potentials at the same physical RE node through their respective virtually shorted feedback loops. Each amplifier corrects any deviation from its programmed bias, and the shared RE is therefore driven simultaneously toward multiple voltages. Both negative feedback loops that establish the virtual short are connected to the same node, leaving the cell potentials undefined and invalidating any SMT measurement.

This phenomenon, in which competing feedback loops conflict in establishing a stable reference potential, is defined here as stability-based interference. Unlike classical electrical crosstalk, which involves direct superposition of one stimulus waveform onto another measurement channel [45,101], stability-based interference originates from closed-loop control conflicts within the biasing circuitry itself. The cell bias is no longer well-defined during parallel measurements, which means that the measurement is invalid and not even post-processing can be used to filter out crosstalk. This result is particularly striking because the GWE extension is the most obvious design choice for SMT, given that the GWE potentiostat is widely used in commercial instrumentation. Importantly, as shown in Table 1, this is a unique failure mode for instruments operating under SMT conditions and represents a key design consideration that needs to be fully understood for effective SMT measurements.

#### 5.2.2. Bipotentiostats and Multi-WE Systems in Shared Electrolytes

**Figure 10:**
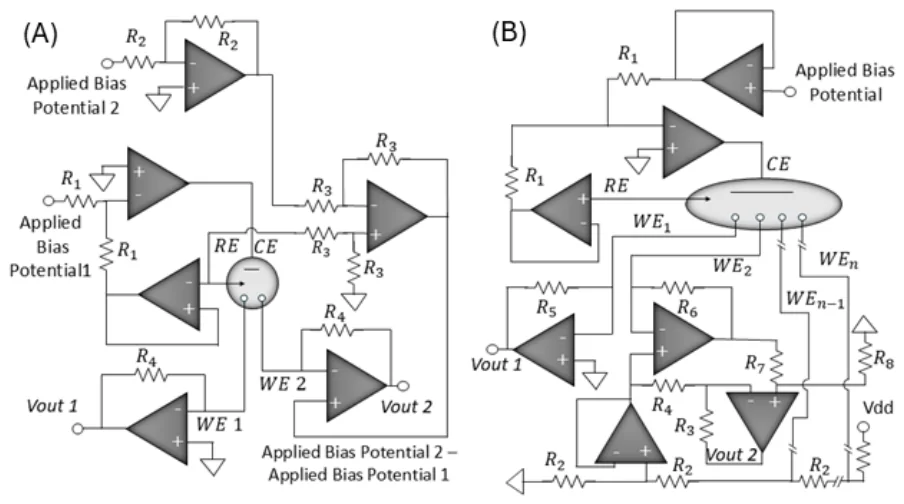
Multi-WE potentiostat architectures derived from the GWE topology. (**A**) Bipotentiostat with two independently biased working electrodes sharing a single RE and CE. (**B**) Matsue-style multichannel potentiostat with multiple WEs biased through a resistor ladder sharing a single RE and CE pair.

The instability of direct GWE replication can be avoided without abandoning the GWE biasing structure entirely. Two related multi-working-electrode architectures achieve this by sharing a single reference and counter electrode among all working electrodes, so that only one biasing feedback loop is ever closed. The first is the bipotentiostat, often used for rotating ring-disk electrode (RRDE) experiments, in which two working electrodes share a single reference and counter electrode. The bipotentiostat circuit, shown in Figure 10A, retains the GWE biasing structure but adds a second working electrode connected to its own independent transimpedance amplifier [33]. Both working electrodes are referenced to the same RE and supplied by the same CE, with each WE held at an independently commanded potential through its dedicated TIA. The second architecture, shown in Figure 10B, is the Matsue-style multichannel potentiostat [80], which generalizes the bipotentiostat to an arbitrary number of WEs using a resistor ladder. Here, all WEs share a single RE and CE pair, and each WE is connected to its own TIA channel for independent current readout.

Both architectures avoid the stability-based interference described in Section 5.2.1 because there is only one biasing feedback loop closing through the shared RE, and all working electrodes reference the same commanded potential rather than competing for control of independent reference nodes. The single feedback loop eliminates the conflict between bias amplifiers that arises when the GWE topology is directly replicated. Both architectures remain fully exposed, however, to volume–conductor coupling through the shared electrolyte, since two WEs driven at different potentials in the same solution still produce a potential gradient across the inter-electrode solution resistance, and current flows between them along paths that bypass the intended single-cell measurement loop.

This coupling has been thoroughly documented in RRDE experiments, where the disk and ring operate as independently biased working electrodes within a bipotentiostat configuration [99,101]. Dual fast CV experiments at the disk and ring routinely exhibit solution-resistance-mediated crosstalk whose magnitude increases with scan rate and decreases with solution conductivity [43]. A bipotentiostat has also been used to perform simultaneous fast-scan CV for dopamine and chronoamperometry for oxygen in vivo, with electrode spacing and fabrication details specifically emphasized to mitigate capacitive and solution-mediated coupling [108]. This approach works for two WEs but scales poorly as more channels are added, since each additional WE introduces new solution-resistance coupling paths to every other WE in the array.

Larger-scale multichannel implementations confirm this scaling limitation. A 24-channel bipotentiostat for microfluidic platforms has been demonstrated, but the system places each cell in its own physically isolated reservoir to avoid shared-electrolyte coupling [111]. This strategy eliminates inter-cell coupling by construction, but it also eliminates the shared microfluidic channel itself, causing the required sample volume, fluidic complexity, and device footprint to scale linearly with channel count. This outcome is incompatible with miniaturized wearable and point-of-care deployment. The Matsue-style potentiostat topology, in addition to exhibiting electrical crosstalk between adjacent WEs, satisfies only half of the SMT measurement requirements. Its resistor ladder generates multiple bias potentials, which partially fulfills the definition of SMT operation. However, because only a single voltage source drives the ladder, the topology is restricted to applying one technique at multiple potentials. In this respect the resistor ladder represents a step back from the bipotentiostat design, as the distinct stimulus waveforms required for an adjacent CA and CV measurement (or any other pair of dissimilar techniques) cannot be produced from a single resistive ladder [80].

Some high-density multiplexed systems instead use per-cell sample-and-hold capacitors to maintain the applied potential between multiplexing slots [112,113]. These systems conceptually decouple the potentiostat from the cell during the hold period, eliminating inter-channel feedback conflict but introducing potential drift caused by faradaic and double-layer charging over the hold interval. The cyclic voltammograms reported in [112] show noticeable deviations from commercial-potentiostat benchmarks consistent with this drift, indicating that such architectures trade one problem, feedback coupling, for another, bias accuracy.

In summary, bipotentiostat and Matsue-style architectures resolve the stability problem of the direct GWE extension by collapsing multiple feedback loops into one, but they do so at the cost of preserving the underlying solution-resistance coupling that drives electrical crosstalk in shared electrolytes. They are therefore suitable for low-channel-count systems with well-spaced electrodes, or for systems with physically isolated reservoirs, but they do not provide a path to high-density SMT operation in a continuous microfluidic channel. This separates two distinct failure modes that must each be addressed for SMT operation: the stability-based interference of the GWE extension, and the electrical crosstalk that persists through the shared solution resistance. The bipotentiostat and Matsue topologies eliminate the former by sharing a single reference and counter electrode, yet this very sharing is what leaves the inter-electrode solution resistance, and therefore the electrical crosstalk, intact. A complementary strategy is to preserve a full, independently controlled three-electrode cell at every site while holding one of the non-working electrodes at a common analog-ground potential across the array.

#### 5.2.3. Grounded-RE Extensions

Stability-based interference in the GWE extension arises from competing feedback loops fighting for control of electrolyte-coupled RE nodes. One mitigation is to remove the competition by holding all REs at a common analog-ground potential, which corresponds to the GRE topology (Section 3.2). In the multi-cell GRE configuration, each channel’s control amplifier drives its own RE toward the same fixed analog-ground potential. Because all amplifiers command the same RE potential, no conflict arises, and the inter-channel voltage difference at the RE nodes is limited to the few-millivolt level set by OPA offset, which is insufficient to drive measurable current through the inter-cell solution impedance [2,34].

Experimental demonstrations of multichannel GRE potentiostats confirm that this architectural change eliminates stability-based interference with both in-cell quasi-REs and shared external Ag/AgCl REs [2]. In these experiments, a chronoamperometry cell held at 0 V correctly reports zero current while an adjacent cell executes a CV scan from 0 to 0.8 V at 100 mV/s, and the bias on both cells remains well-defined throughout. Holding the CA channel at 0 V isolates the effect of crosstalk from the adjacent CV measurement, but it also limits the ability to observe any destructive interference that may arise when both channels run active electrochemical reactions, which represents an additional limitation of the experimental setup. Critically, although the GRE topology removes stability-based interference, it does not by itself suppress electrical crosstalk: even with the bias well-defined, the CA channel’s current partly tracks the CV current waveform on the neighboring channel, achieving a minimum coupling of approximately 8% when a shared external Ag/AgCl RE is used and adjacent cells are spaced 2.68 mm apart [2]. This residual coupling is the shared volume–conductor crosstalk described in Section 4.2.2, and the reliance on an external macroscale Ag/AgCl RE together with a cell pitch of nearly 3 mm makes the demonstrated configuration unsuitable for the channel densities required by miniaturized lab-on-chip platforms.

The GRE topology therefore advances beyond the bipotentiostat by supporting a full, independently controlled three-electrode cell at every site while still eliminating stability-based interference, but it confirms that solving the instrumentation stability problem is necessary yet not sufficient: the electrical crosstalk mediated by the shared solution resistance remains. This motivates examining whether a different choice of grounded electrode, combined with deliberate electrode geometry, can begin to address the residual coupling.

#### 5.2.4. Grounded-CE Extensions

The most successful, although still limited, demonstrations of true SMT measurements have employed the GCE topology [114,115]. In a multichannel GCE configuration, the CE of each cell is held at the same analog-ground potential, which, by the same argument used for the multichannel GRE, eliminates CE-to-CE potential differences and the associated stability-based coupling. What distinguishes these demonstrations from the GRE results above, however, is not the choice of grounded electrode alone but the fact that they begin to reduce electrical crosstalk as well. Giagkoulovits et al. [114] demonstrated a 16×16 CMOS microelectrode array using the GCE topology with adjacent chronoamperometry channels, and Li et al. [115] demonstrated a 128-channel GCE array with localized CE rings around each WE. Both implementations report low crosstalk between adjacent channels, but the success of these systems is not attributable to the GCE topology alone; rather, the electrode geometries used in both cases incorporate guard structures that locally terminate field lines between adjacent cells. This points to electrode geometry, and specifically the guarding effect introduced by local CE structures, as a key enabler of SMT operation that complements the choice of potentiostat topology. The guard-ring geometries used in these systems, and the broader role of electrode-level shielding in suppressing inter-cell coupling, are examined in the following section.

### 5.3. Guard Rings and Local Shielding

A fundamentally different approach to crosstalk mitigation is to modify the electrode geometry itself so that a guard structure is placed between every pair of adjacent cells. The guard is a conductor held at a common, stable potential that terminates the electric field lines propagating from one cell and thereby shields neighboring cells from the disturbance. Conceptually, this is identical to the guard-ring technique used in high-impedance analog circuit design.

**Figure 11:**
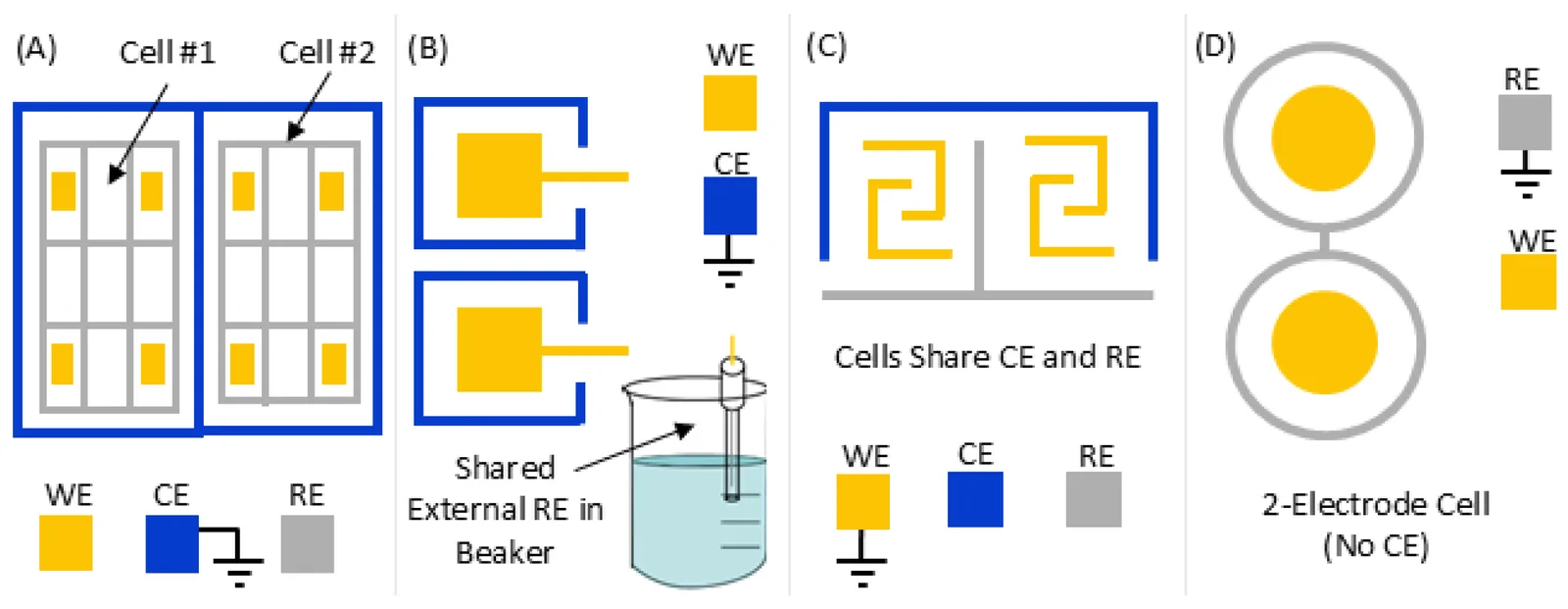
Representative three- and two-electrode geometries used for crosstalk suppression in SMT electrochemical arrays. (**A**) Matrix-style WE cluster with surrounding RE matrix and outer CE ring, adapted from [114]. (**B**) Individual WEs each encircled by a local CE ring with a shared external Ag/AgCl RE, adapted from [91,115]. (**C**) Shared CE and RE with interdigitated WEs, adapted from [116]. (**D**) Two-electrode cells with a common RE wrapped around each WE, adapted from [45].

Four representative geometries that have been reported in the SMT electrochemistry literature are summarized in Figure 11. Giagkoulovits et al. [114] present a 16×16 CMOS microelectrode array in which four WEs forming a single cell are surrounded by a ring of RE segments, and the group is in turn enclosed by a common CE ring (Figure 11A). Because all CE rings across the array are held at the same potential by the GCE topology, the outer boundary of every cell is at a common potential, and current flow between cells is largely confined through the ring CE rather than into neighboring cells. Detailed finite element analysis supports this picture [114], and the authors report approximately 12% crosstalk between adjacent chronoamperometry measurements. Li et al. [115] and Hasegawa et al. [91] use a related geometry in which each WE is directly encircled by a Pt CE ring, with a shared external Ag/AgCl RE referencing all cells (Figure 11B). Both report essentially crosstalk-free operation for 128-channel and smaller arrays. In ref. [116], electrode geometry is constructed to allow individual WEs in a dense array to share a common CE and RE pair (Figure 11C). This geometry suppresses chemical crosstalk through separation but constrains the system to a single electrochemical control loop and therefore does not support SMT. Finally, ref. [45] describes a two-electrode architecture in which a common RE wraps each WE (Figure 11D) and demonstrate that unguarded coupling extends up to 1 mm, while the wrapped-RE geometry reduces the detectable coupling distance to approximately 50 μm.

These geometries make a strong case for the role of common-potential guarding in suppressing both chemical and electrical crosstalk. Two important caveats appear when this literature is examined closely. First, the reference electrode choice confounds the attribution of improvement to geometry. Both refs. [115] and [91] use a shared external Ag/AgCl RE rather than microfabricated REs integrated in the array. Lobert et al. [2] showed in a controlled comparison that replacing microfabricated Ti/Au quasi-REs with a shared external Ag/AgCl RE substantially reduced crosstalk in a GRE potentiostat without changing the electrode geometry. This result implies that a substantial fraction of the crosstalk reduction observed in [91,115] may be attributable to the shared nonpolarizable RE rather than to the local CE ring geometry alone. External macroscale REs are bulky, non-ideal for wearable and microfluidic deployment, and introduce a single point of failure in the fluidic path [110,117,118,119]. Miniaturized in-channel Ag/AgCl REs fabricated by chemical oxidation or anodization are preferable but have limited shelf life due to AgCl depletion and are susceptible to degradation by chloride-mediated Au dissolution in mixed-material arrays [120,121]. A rigorous design framework for SMT sensors must therefore decouple the role of guard geometry from the role of RE choice.

Second, even if their systems are SMT capable, most of the works above demonstrate guard effectiveness under a single electrochemical technique applied across all cells, such as chronoamperometry in [114,115] or voltage recording in [45], or with very specific potentiostat topologies integrated on-chip. They do not test the case of conflicting stimulus waveforms, such as CV simultaneous with CA, across adjacent cells, which is the defining condition of SMT. As the next section shows, this is the case in which the interaction between potentiostat topology and guard electrode choice becomes critical.

A third qualification concerns the scope of what guarding achieves. A common-potential boundary suppresses the volume–conductor coupling that arises from inter-cell potential gradients, but it does not prevent diffusional overlap or the transport of reaction products between adjacent cells, does not resolve instrumentation ground loops, and does not eliminate parasitic capacitive coupling between signal leads, which is set by routing rather than by the in-solution electrode geometry. Guarding is therefore necessary but not sufficient for interaction-free SMT operation.

Table 2 summarizes representative multi-cell platforms alongside their potentiostat topology, guard electrode, operating mode, and reported inter-cell crosstalk. Both the choice of potentiostat topology and the use of a guard-ring geometry show varying degrees of success in suppressing crosstalk across these systems, and neither factor alone reliably predicts the reported coupling. Instead, the platforms reporting the lowest crosstalk are those in which an appropriate grounded-electrode topology is paired with a guard structure that holds the boundary of each cell at a common potential. This recurring pairing indicates that successful SMT measurement depends on the combination of instrumentation and electrode geometry rather than on either in isolation.

## 6. A Co-Design Framework for SMT Sensor Arrays

Section 5 shows that each existing strategy addresses a subset of the coupling mechanisms in shared-electrolyte arrays but leaves others unaddressed. Bipotentiostats eliminate feedback conflict but remain exposed to shared volume–conductor coupling. The GWE extension introduces stability-based interference, the GRE extension leaves residual shared-electrolyte coupling, and the GCE extension fixes the common-potential boundary but ties the readout node to the guard. Guard rings suppress lateral coupling, but their effectiveness depends on the potentiostat topology that drives them. Taken together, these findings suggest that the achievement of interaction-free SMT operation in a microfluidic channel therefore requires that the potentiostat circuit and the three-electrode cell geometry be co-designed to satisfy three principles simultaneously. The framework developed below is an analytical synthesis of the circuit behavior established in Section 3 and the outcomes reported in Section 5, rather than an experimentally validated design rule.

### 6.1. Three Principles for Interaction-Free SMT Operation

Applying the three principles to sensors sharing a single, electrically connected solution yields three potentiostat configurations compatible with interaction-free SMT operation. The first principle (P1) for interaction-free SMT operation is that adjacent cells must be separated by a guard electrode held at a stable, common DC potential across the array. When the boundary of every cell is at the same potential, no voltage gradient develops across the inter-cell solution resistance, and the dominant shared volume–conductor coupling path identified in Section 4.2.2 is suppressed. This principle is consistent with the guard-ring geometries surveyed in Section 5.3, in which CE rings of the GCE topology or RE structures of the GRE topology serve as the common-potential boundary.

The second principle (P2) is that the guard cannot be the working electrode. As the active sensing site, the WE is generally sized as small as practical to maximize resolution and limit-of-detection. However, the guard ring should be physically large to provide a common isolating potential among all cells. These competing geometric and electrical requirements make the WE fundamentally unsuited as a guard, leaving only the RE or the CE.

The third principle (P3), which has not been articulated explicitly in prior work, is that the guard must be driven from a low-impedance source. This principle explains why the conventional TIA-based GCE topology (Figure 5A) underperforms in SMT operation even when the first two principles are satisfied. In that topology the CE is held at a virtual ground through the feedback loop of the transimpedance amplifier, with the CE connected to the inverting input and a feedback resistor. While the virtual short condition nominally fixes the CE at ground, a large feedback resistor required for sensitive current measurement raises the closed-loop output impedance at the CE node and, more importantly, makes the CE the current-sensing node itself. Small currents injected into the CE by neighboring cell activity therefore both perturb the CE potential and are read directly through the feedback resistor as measured current, so the guard electrode and the crosstalk-injection node are one and the same. The solution is to decouple the guard from the readout, allowing the CE to be driven by a low impedance source to allow its potential to be stable. This is precisely the physically grounded GCE variant introduced in Section 3.3 (Figure 5B), where the CE is tied directly to a ground rather than to a TIA input and current is, instead, measured at the WE [83,84]. The same low-impedance condition is realized in the CMOS implementations of Giagkoulovits et al. [114] and Li et al. [115], where a DAC and ADC control the WE-to-RE potential difference. This explains why minimal crosstalk was observed in these two examples where the CE is connected directly to a physical ground, making the CE a stiff low-impedance node and enabling effective guarding.

### 6.2. Ideal Potentiostat Configurations for SMT

**Figure 12:**
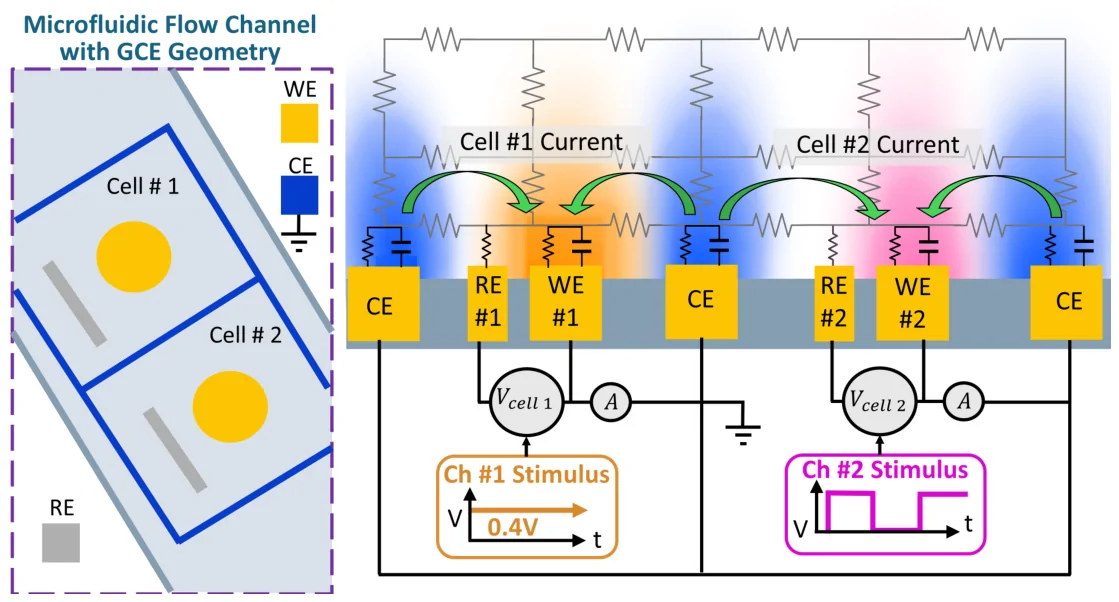
Example SMT sensor utilizing a GCE potentiostat and complementary electrode geometry following the co-design framework. A shared, grounded CE fully surrounds each WE–RE pair, isolating each cell’s current and preventing a potential gradient between adjacent cells that would otherwise produce crosstalk. The GCE potentiostat with current readout at the WE complements this geometry to enable effective SMT measurements.

The first configuration is the modified GCE topology in which the CE is connected directly to a physical ground reference and current readout is performed at the WE rather than at the CE, corresponding to the GCE variant of Figure 5B. Figure 12 illustrates how this topology is paired with a complementary electrode geometry to realize an optimal co-designed array. This configuration satisfies all three principles. The shared CE provides a common-potential boundary to avoid potential gradients from forming between adjacent cells, current readout is performed at the WE, and the physical ground connection makes the guard a low-impedance node. The CMOS implementations of Giagkoulovits et al. [114] and Li et al. [115] realize this configuration through DAC-controlled WE-RE biasing with the CE physically grounded, and both report low inter-channel crosstalk. This configuration carries the strongest experimental support of the three. In both demonstrations, however, the adjacent channels either execute the same technique at different potentials or drive only one cell while measuring crosstalk on an adjacent unstimulated cell. The configuration has therefore not yet been tested under the full SMT condition with multiple analytes of interest.

The second configuration is the GRE topology with a RE-based guard ring shared across all cells. The RE is held at analog ground by a control amplifier that closes its feedback loop through the cell electrolyte rather than through a high-value feedback resistor. At high solution conductivity, the effective source impedance at the RE node is much closer to that of a unity-gain buffer than to that of a TIA, satisfying the third principle. Because the guard is driven through the solution resistance rather than by a direct connection, however, this condition is conductivity dependent, and P3 is satisfied only where the supporting electrolyte is sufficiently conductive. Current readout is performed at the physically separate WE through a dedicated TIA. The experimental GRE demonstration of Lobert et al. [2] is instructive precisely because it satisfies the first principle in its instrumentation but fails to consider electrode geometry. Here, the GRE topology holds every RE at a common analog ground, yet the array contained no dedicated inter-cell guard electrodes and residual crosstalk remained the dominant artifact in SMT measurements. The separation of an effective circuit topology from an incomplete geometry is direct evidence for the necessity of co-design.

The third configuration is the bipotentiostat extended with a matrix-style RE and CE pair that locally surrounds each WE. Its practical advantage over the other two configurations is maturity, as multichannel bipotentiostat instruments are commercially available and do not require custom system design which would be required for the other topologies. The bipotentiostat differs from the GWE and GCE configurations in that it does not implement independent three-electrode cells. Instead, one WE is held at analog ground while the RE is actively driven to establish the cell potential, and each additional WE is held at a virtual ground referenced to the same actively driven RE. This shared biasing structure satisfies the third principle by construction, since a single control loop drives the shared RE and no competing feedback paths exist. However, the absence of independent RE and CE structures per cell means that the first principle, the common-potential guard boundary, can only be satisfied through electrode geometry rather than through additional instrumentation, since one RE and one CE must control the potential gradients across every cell in the array. A matrix arrangement in which a localized RE segment and a CE ring directly surround each WE could, in principle, establish each cell potential locally and minimize the potential gradients through the global electrolyte that drive shared-electrolyte crosstalk. Moreover, the absence of independent three-electrode cells introduces additional considerations regarding chemical crosstalk and reference electrode stability that do not arise in the first two configurations. This configuration, therefore, requires additional investigation before it can be deployed in practice. Table 3 summarizes how the co-design framework relates to extending each of the potentiostat topologies and identifies those most likely to enable successful SMT measurements in a shared volume–conductor.

Taken together, these configurations indicate that true SMT operation in a single shared microfluidic channel may be attainable without resorting to time-division multiplexing or physically partitioned reservoirs, the two compromises that have historically constrained continuous multi-analyte microfluidic sensing. These configurations are identified by circuit analysis rather than by controlled experiment. Because no reported platform varies potentiostat topology and guard geometry independently within the same array and electrolyte, the low crosstalk reported in [114,115] cannot presently be attributed to the grounded-CE topology, to the local CE ring, or to the shared external RE in isolation. By identifying the potentiostat–geometry pairings that are predicted to suppress crosstalk while preserving a single continuous electrolyte, the co-design framework narrows the design space that must be explored experimentally to remove this barrier.

## 7. Conclusions

This review examined simultaneous multi-technique electrochemical sensing in shared-electrolyte microfluidic arrays from a combined instrumentation and electrode geometry perspective. Traditional potentiostat topologies were surveyed and assessed for multi-cell operation, and the chemical and electrical crosstalk mechanisms arising in shared electrolytes were classified, including a stability-based interference mode identified here as a distinct instrumentation-level category. A survey of reported multi-cell platforms showed that effective crosstalk suppression depends jointly on circuit topology and electrode geometry rather than on either in isolation.

From this analysis, a co-design framework was synthesized around three principles: adjacent cells must be separated by a guard electrode held at a common DC potential, the guard cannot be the working electrode, and the guard must be driven from a low-impedance source. These principles identify three potentiostat configurations capable of interaction-free SMT operation. This review unifies potentiostat topology and electrode geometry within a single coupled design space, and the resulting framework both prescribes these candidate configurations and retrospectively explains the crosstalk outcomes of previously reported platforms.

Realizing these configurations on a shared hardware platform, where topology and electrode geometry can be varied independently under fixed measurement conditions, is the immediate next step and would convert the three principles into quantitative design rules. Establishing SMT operation on such a platform also reframes problems that are presently inaccessible. Parallel measurement makes the effect of a design change observable across channels, so electrode-layout optimization and circuit-parameter selection become tractable design problems, and the inter-cell coupling characterized qualitatively here can be measured systematically enough to support crosstalk prediction and compensation. Multichannel signal decoupling becomes meaningful only once channels are driven independently, since a multiplexed array produces no simultaneous mixture to separate. The same holds for the emerging technologies that continuous multi-analyte sensing will require. Integrated Lab-on-CMOS platforms gain their advantage in routing and per-cell guard density only when every cell is instrumented in parallel. Adaptive instrumentation presupposes concurrent channels whose interaction can be sensed and acted upon. AI-assisted electrochemical sensing depends on the parallel multi-analyte data streams that only SMT operation produces, whether for sensor-drift correction or for adaptive measurement across an array. In each case, interaction-aware SMT instrumentation is the enabling technology.

## Figures and Tables

**Table 1:** Crosstalk susceptibility of electrochemical array operating modes. A check mark (✓) indicates that the mechanism is a challenge that must be managed when using that mode while a cross (✗) indicates the mode is not susceptible. Multiplexed operation avoids all mechanisms by activating a single cell at a time while SMT represents the most challenging operating condition.

Crosstalk Mechanism	Cause	Crosstalk Susceptibility		
		Mux	SST	SMT
Chemical crosstalk	Diffusion layer overlap	✗	✓	✓
Instrumentation ground loops	Shared instrumentation ground connections	✗	✓	✓
Capacitive coupling	Parasitic capacitance between adjacent electrodes/leads	✗	✓	✓
Volume–conductor coupling	Current paths through shared conductive electrolyte	✗	✓	✓
Stability-based interference	Independent per-cell bias setpoints	✗	✗	✓

**Table 2:** Reported multi-cell electrochemical platforms classified by instrumentation topology, presence of an inter-cell guard electrode, degree of simultaneity, and reported inter-cell crosstalk. Multiple potentiostat topologies and electrode geometries have been implemented with varying levels of successful crosstalk mitigation.

System	Topology	Guard	Mode	Reported Crosstalk
Giagkoulovits et al. [114]	GCE	CE	SMT	12%
Li et al. [115]	GCE	CE	SMT	Below circuit noise floor
Lobert et al. [2]	GRE	No	SMT	8%
Vergani et al. [111]	Bipotentiostat	No ^†^	SMT	N/A (isolated reservoirs)
Matsue et al. [80]	Multi-WE resistor ladder	No	SMT ^‡^	Present but unquantified
Tedjo & Chen [116]	GWE	CE and RE	SST	Below circuit noise floor
Naughton et al. [45]	Two-electrode	RE	SST	25% at 50 μm spacing

^†^ Coupling avoided by physically isolated per-cell reservoirs rather than a guard electrode. ^‡^ Multiple potentials, single technique (partial SMT).

**Table 3:** Evaluation of potentiostat topologies against the three co-design principles. Each topology is paired with its grounded electrode acting as the inter-cell guard. Violation of any principle disqualifies the configuration from interaction-free SMT operation. Topologies requiring future validation are listed as RFV.

Topology (Guard)	Advantage	Limitation	SMT Capability and Evidence
GWE (WE guard)	None identified	Violates P2 & P3; Circuit instability.	Not SMT capable [2]
GRE (RE guard)	Requires only a single RE per array	P3 only satisfied at high solution conductivity	SMT capable (RFV)
GCE, TIA-based virtual ground (CE guard)	Large CE is ideal for guard ring geometry	Violates P3	Not advised for SMT
GCE, physically grounded CE (CE guard)	Large CE is ideal for guard ring geometry	None identified (RFV)	SMT capable supported by [114,115]
Bipotentiostat	Multichannel instruments are commercially available	Shared RE and CE constrain geometry	SMT capable (RFV)

## Data Availability

The original contributions presented in the study are included in the article; further inquiries can be directed to the corresponding author.
